# A review of the foliar application of individual amino acids as biostimulants in plants

**DOI:** 10.1007/s44279-025-00222-7

**Published:** 2025-05-13

**Authors:** Bethany C. R. Henderson, John M. Sanderson, Andrew Fowles

**Affiliations:** 1 Department of Chemistry, Durham University, Durham, UK; 2 Croda Europe Ltd, Snaith, East Cowick, East Yorks, UK

**Keywords:** Amino acid, Antioxidants, Biochemistry, Biostimulant, Chlorophyll, Crop yield, Foliar application, Oxidative stress, Photosynthesis, Photosystem II (PSII), Sustainable agricultural practices

## Abstract

Enhancing crop yields in an environmentally sustainable manner is crucial in the agricultural sector, given the challenges posed by population growth, climate change, and the need to reduce pesticide usage. Biostimulants, such as amino acids, offer natural and stable solutions to improve yield, growth, and resistance to external factors. They achieve this by reducing the impact of abiotic stress and stimulating plant defence mechanisms. As biostimulants, amino acids' simplicity, cost-effectiveness, and environmental friendliness have garnered recognition whilst offering natural and stable solutions to improve crop yield and resistance to external factors. Mixtures containing multiple amino acids are commonly used in agriculture, but the effects of individual components within these mixtures are less understood. This review summarises current knowledge on the effects of the foliar application of the 20 most common naturally occurring amino acids. The similarities in observed effects, as well as proposed modes of action, have been discussed. Notably, distinct differences in the modes of action of these amino acids have been observed, with two key themes emerging: firstly, an enhancement in the rate of photosynthesis through the promotion of chlorophyll synthesis or improved electron transfer in Photosystem II (PSII); secondly, protection against oxidative stress, either by increasing antioxidant synthesis or directly acting as antioxidants. For some amino acids, the mode of action after foliar application is unclear, or the effects are minor, highlighting the need for further research into their specific mechanisms of action.

**Supplementary Information:**

The online version contains supplementary material available at 10.1007/s44279-025-00222-7.

## Introduction

As the global population continues to increase in size and the demand for food increases, the need to improve plant crop yield is becoming increasingly urgent. It is estimated that to combat the deficit between production and need by the year 2050, global agricultural output must increase by 60% [1]. In parallel to population increase, climate change increases environmental stresses in areas where crops are produced. Therefore, interest in the use of biostimulants for the promotion of valuable traits in agricultural crops, such as improving yield and resistance to adverse environmental factors, has increased rapidly over the past decade [2]. Biostimulants may offer an environmentally conscious improvement to the discrepancy between demand and availability in conjunction with other current methods for improving yield. Amino acids are promising biostimulants as they are abundant natural products that plants can safely metabolise and are environmentally benign.

The term biostimulant refers to any substance or microorganism externally applied to plants to improve nutrition efficiency, stress tolerance or crop quality traits. These compounds are usually derived from natural sources, are used in small amounts and can be categorised into a number of different groups. These groups include humic and fulvic acids (found in soil), seaweed and plant extracts (containing nutrients and plant hormones to stimulate metabolism), inorganic compounds (such as phosphites and silicon), chitosan and other biopolymers (derived from plant/animal sources), and amino acids (found naturally in plants for protein synthesis) [3].

Research into biostimulants has greatly increased in recent years, with over 700 papers published on “plant biostimulants” between 2009 and 2019 [4]. This number has increased even more in recent years, with 100s of papers published every year on this topic [5]. Amino acids are a group of compounds already used in agriculture as biostimulants, usually as mixtures. Application of many amino acids has been shown to increase the yield of crops and also reduce the effects of stresses on plants, and so improve growth in less desirable environmental conditions, such as where soils are saline or where heat or drought stress is present [6]. Despite the extensive number of papers looking at amino acids as biostimulants, a significant gap remains regarding the comparative and mechanistic analysis of amino acids as biostimulants. The need for a comprehensive overview of current research is critical in understanding trends between different amino acid types.

Of the 23 proteinogenic amino acids, 20 are encoded by the nuclear genes of eukaryotes [7] and all (excluding glycine) have an *L*-α-configuration [8]. As the three non-nuclear encoded amino acids (Selenocysteine, Pyrrolysine and N-Formylmethionine) are not standard cases (Sec being incorporated via tRNA, Pyl being used in some bacteria and archaea, and fMet being the form of Met used to initiate protein synthesis in prokaryotes), these have not been included in this review.

Foliar application of amino acid mixtures such as ‘Amino16®’, which contains an 11.3% mixture of amino acids including all the aliphatic, basic, acidic and hydroxylic amino acids alongside methionine, phenylalanine, and tyrosine, has been shown to improve plant yield in several plant species, including lettuce and olive trees (*Olea europaea var. minor rotunda)* [9, 10] and are already used within industrial agricultural practices. Some research into the mechanisms of amino acid mixtures has been done. Li et al. reported that after the foliar application of an unspecified mixture of water-soluble amino acids, almost 4000 differently expressed genes (DEGs) were up-regulated, while the down-regulation of almost 3000 DEGs was observed. These genes were primarily involved in photosynthesis, photosynthesis-antenna proteins, plant-pathogen interactions, phenylpropanoid biosynthesis, glyoxylate and dicarboxylate metabolism, Mitogen-Activated Protein Kinase (MAPK) signalling pathway and plant hormone signal transduction [11]. However, due to the presence of multiple amino acids in these supplements, it is not possible to determine if they were all necessary to reach the desired effect and, if so, how each interacted within the plant to cause these effects.

Understanding how these amino acids interact with plants to affect plant health is necessary for exploiting their beneficial qualities further. Foliarly applied amino acids enter the leaves through negatively charged, hydrophilic pores, which travel across the leaf’s waxy cuticle [12]. Below pH 9, the -amino group is in the positively charged ammonium form, and the amino acid readily moves through these pores. In addition to this effect of amino acid charge, amino acids with hydrophobic side chains are able to diffuse into the leaf more readily than those with hydrophilic side chains using diffusion forces [13].

In some cases, the amount of amino acid applied to the plant has been found to play a role in the effect on plant yield. For example, the application of L-methionine to lettuce (*Lactuca sativa* L.) produced an inverse effect on growth at high concentrations, contrasting to an increase in yield at lower concentrations, leading to a suggestion that over-stimulation damages the photosynthetic apparatus [14].

This review aims to summarise the currently available knowledge of how amino acids impact plant growth and to look for similarities and differences between both different classes of amino acids and individuals within the same class.

## Current research on foliar application of amino acids

There are a number of ways to categorise the 20 common amino acids, but for the purposes of this review, they are classified based on the structure of their side chains into seven groups: aliphatic, aromatic, acidic, basic, hydroxylic, sulfur-containing, and amidic. This classification was chosen as the mechanism of action is often class-specific.

### Controls and conditions

To evaluate the correlation between the foliar application of amino acids and the proposed effects on plants, it is crucial to consider the controls employed to support these assertions. Additionally, it is essential to examine the growing conditions utilised in the plant trials. In the papers discussed in this review, there are several themes in the controls used. Some papers report extensively on their control treatments and the conditions in which their trials occurred, while others provide fewer details. Full details of the controls in each paper are available in the supporting information.

#### Treatment of control plants

In the case of the papers included here, these control treatments were either:

- no treatment given, which provides a comparison that is true to the natural growth conditions without any additional treatments but still allows for doubt regarding the specific ingredient (e.g. surfactant) in the foliar spray responsible for the effects;
- water, which uses distilled water in most cases, although tap water is sometimes used, which has the same issue as a) concerning uncertainty over the active component and
- treatment with the same composition of spray but with amino acid removed (i.e. still containing surfactants and other adjuvants), which eliminates the possibility that something other than the amino acid is causing the observed effects

#### Growth conditions

An imperative consideration in the comparative analysis of studies of the efficacy of foliar sprays involves an examination of the environmental parameters governing plant growth. Ideally, studies should document the minimum and maximum temperatures the plants encountered, along with details about soil composition and humidity levels. Additionally, the timing of experiments holds significance, as it has been demonstrated that the season in which trials are conducted can influence the growth of the plants and, therefore, add another level of uncertainty as to the effects of the biostimulant [15].

### Aliphatic amino acids

The aliphatic amino acids (Alanine, Glycine, Isoleucine, Leucine, Valine and Proline) can all be classed as non-polar based on their hydrophobic side chains (except for glycine).

There is no consistent theme for the activities of exogenously applied aliphatic amino acids. However, there are a few similarities between some of them [13]: proline and alanine both contribute to the plant’s antioxidant defence system, proline works directly as an antioxidant, and alanine promotes an increase in polyphenolic compounds which can act as antioxidants; glycine and proline cause an increase in chlorophyll concentration; the branched-chain amino acids appear to promote the response of plant growth regulators, although more research is needed to determine their exact mode of action. Only proline has been extensively studied in isolation; this amino acid gives the best evidence for a positive effect on growth. In all cases, however, there is a need for better control experiments to rule out effects from other components of the spray.

#### Alanine

Alanine has mainly been studied as part of a mixture with other amino acids, so the actual influence of alanine on plant growth is unknown. A comparative study of foliar application of amino acids to *Eustoma grandiflorum* showed that foliar application of alanine reduced the final dry mass of the plant in comparison to a control of water spray. It also reduced the number of leaves produced and had a negative effect on the yield [16].

However, contrary to these findings, one study has shown a link between the foliar application of alanine and improved quality of some fruit species, specifically Fuji apple (*Malus domestica 'Fuji'*), by increasing the total amount of compounds responsible for aroma and increasing the activity of alcohol dehydrogenase (ADH) and alcohol acyltransferase (AAT) enzymes involved in the metabolism of amino acids [17]. These effects on fruit yield for alanine were significantly greater than that of the effects of foliar application of leucine, isoleucine and valine [18]. However, in both the studies mentioned here, limited information was provided about the control plants used or the conditions in which the field trials took place, which leaves room for doubt over the significance of these effects.

#### Glycine

The effects of glycine vary significantly between plant species, ranging from no effect in some species such as maise (*Zea mays*), [19] to improved growth in others, such as lettuce and sweet basil (*Ocimum basilicum*). In the latter cases, glycine at low concentrations (between 250 mg L^−1^ and 500 mg L^−1^) improved plant growth and several characteristics relating to yield, including chlorophyll concentration, fresh and dry mass, and leaf area [20, 21]. It was suggested that this improved yield may be related to an increase in the concentration of chlorophyll and carotenoids in plant leaves. In the comparative study of amino acids in *Eustoma grandiflorum * [Sect.  2.1.1], [16] glycine was shown to increase chlorophyll levels and produced the greatest increase in plant height despite no significant difference in dry mass being observed. Control plants in all of these studies were treated with distilled water, so there remains some uncertainty over the active ingredient.

As glycine is a precursor for the synthesis of chlorophyll via chlorophyll A, this pathway may operate to increase chlorophyll levels [22]. Khan et al*.* also suggested that an increase in chlorophyll concentration caused by foliar application of amino acids, with a concomitant increase in the rate of photosynthesis, leads to improved plant yield [23].

**Fig. 1:**
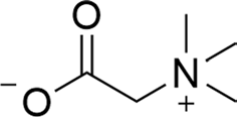
Trimethyl glycine (or glycine betaine)

More extensive research has been done into the application of exogenous glycine betaine (Fig. 1). This molecule has proved to be highly effective when used in a number of different plant species under differing stress conditions, including drought, high salinity, and extreme temperatures [24, 25]. However, in cotton (*Gossypium herbaceum*), glycine betaine did not show improvement against environmental stresses [26].

Glycine betaine can reduce oxidative stress as it increases the expression of antioxidant enzymes (e.g. ascorbate peroxidase) [27]. This effect is also thought to be related to a sequestering of sodium ions in plant roots. Under salt related stress in rice (*Oryza sativa*), application of glycine betaine as a foliar spray yielded lower sodium ion levels in the shoots (a sign of reduced stress) compared to control plants, where individual substrates were omitted from each trial [28].

#### Branched chain amino acids (isoleucine, leucine and valine)

The limited studies on the foliar application of these three branched-chain amino acids have yielded a similar improvement in growth to the soil application of urea, with increased shoot density but no significant difference in root length or in root and shoot weight [13]. However, there was no significant change in the levels of any growth factors compared to untreated plants when these amino acids were applied individually or in pairs. The improved shoot density has been suggested to be related to a plant growth regulator rather than the provision of an additional nitrogen source. However, no suggestion as to which plant growth hormone the action was related to or how the amino acid may interact with these growth hormones was given.

#### Proline

The foliar application of proline is a potentially effective tool for improving yield in poor growing environments affected by climate change [29, 30]. There is debate over whether an increased concentration of endogenous L-proline is directly caused by stress to the plant or as an internal response to the stress, with proline acting as part of the plant’s antioxidant defence system [31]. It has been observed that in plants which are put under stressed growing conditions, there is a greater concentration of proline and a lower yield [32]. However, when L-proline is applied exogenously, positive effects are seen against heat stress, high salinity, and heavy metal induced stress in many plant species, suggesting that the internal response to stress is the critical factor responsible [33–35]. The positive effect proline application has on environmentally stressed plants may be linked to antioxidant effects, scavenging reactive oxygen species (ROS) to form adducts of proline derivatives such as 4-hydroxyproline and 3-hydroxyproline as well as the promotion of nitrogen fixation [36, 37]. Mondal et al*.* suggest that proline also offers a source of carbon, nitrogen, and oxygen to the plant to aid its recovery if exposed to stress [16]. However, as this activity is not seen with other amino acids, and the effect is seen with very low concentrations, it seems unlikely that such a small amount of additional proline could produce such beneficial results if acting purely as a source of nutrients.

It has been posited that foliar application of L-proline improves nitrogen fixation at the roots of plants, leading to improvements in the synthesis of amino acids and chlorophyll, leading eventually to increased photosynthesis and improved yield [38]. Interestingly, it is notable in these studies that increased chlorophyll content in the plant was correlated to a low amount of nitrogen in the soil. This may be because proline uses a less favourable pathway for nitrogen fixation than the pathway used in the absence of stress, and so it is only used if the plant is under stress and proline is available.

The studies above all use either water or no treatment on the control plants, so there is room for doubt over whether another component in the mixture is responsible for the effect. However, given the number and diversity of studies reporting growth enhancements, proline clearly has some positive effects. The range of compositions used in these studies are all between 2 and 1600 mg L^−1^.

### Aromatic amino acids

As with alanine in the previous section, foliar application of aromatic amino acids, especially tryptophan, [39] induces an increase in the levels of phenolic compounds within the plant. This response is mediated by an increase in the levels of abscisic acid. Increased levels of phenolic compounds can improve a plant's ability to withstand oxidative stress by providing a sink for ROS.

#### Phenylalanine

**Fig. 2:**
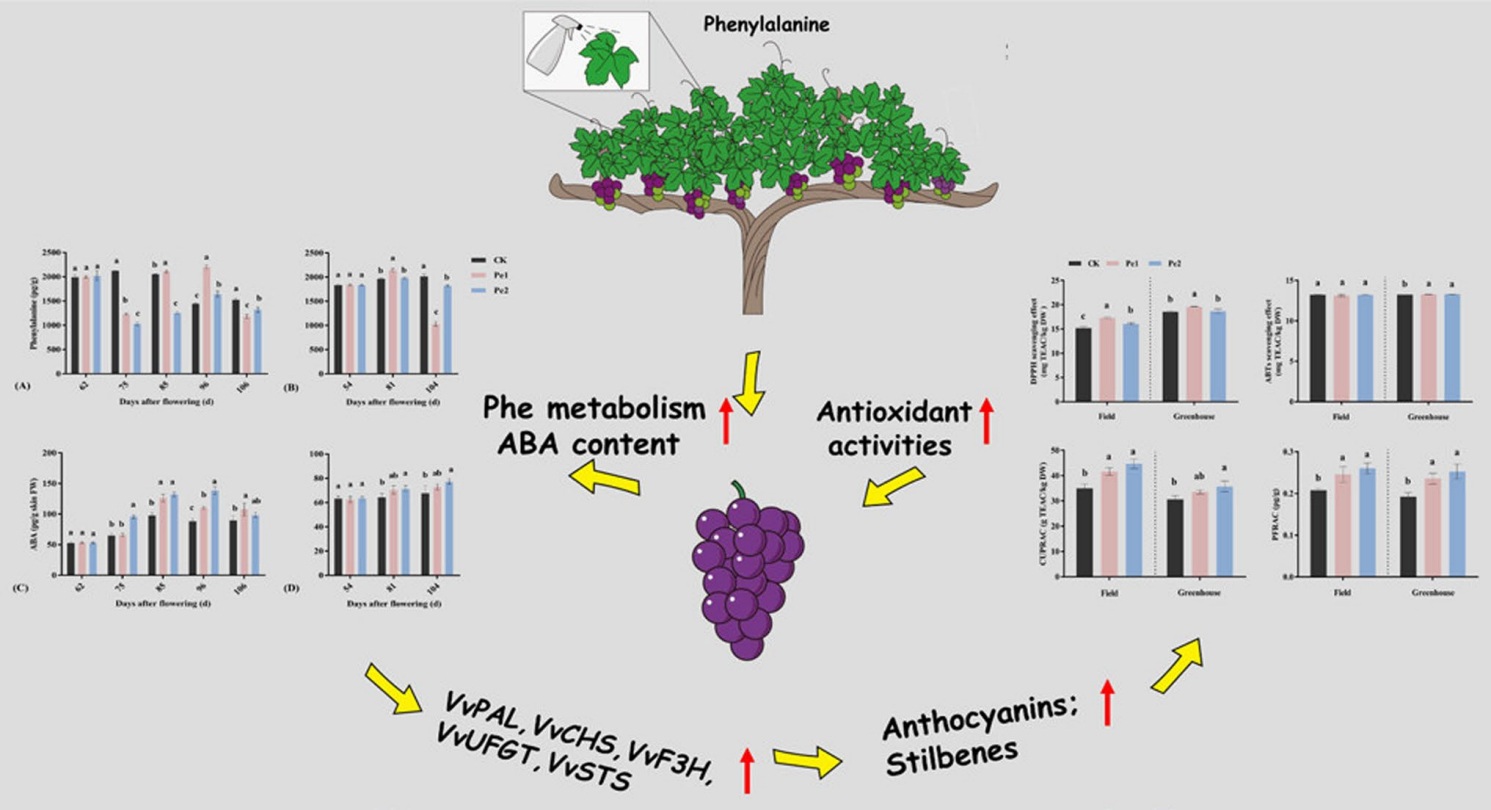
Foliar application of Phe promotes the synthesis of Anthocyanins and Stillbenes through metabolism to ABA, which promotes the expression of genes responsible for polyphenol production. Reprinted with permission from Cheng et al. [45]. Copyright 2020 American Chemical Society

Most research into the foliar application of phenylalanine has looked at its ability to increase the levels of phenolic compounds (anthocyanins and stilbenes) in grapes (*Vitis vinifera*), which is beneficial for improving quality in wine production. The application also increases the synthesis of some amino acids within the grapes, particularly phenylalanine itself, without changing the total amino acid content when compared to control plants with phenylalanine omitted [40–44]. Increased levels of phenolic compounds lead to improved antioxidant activity in grape vines grown in low-nitrogen environments [45–47]. This increase occurs as the increase in phenylalanine leads to an increase in abscisic acid (ABA) levels. These increased ABA levels upregulate the expression of some genes (*VvPAL, VvCHS, VvF3H, VvUFGT,* and *VvSTS*) in the phenolic synthesis pathway (Fig. 2) [45].

Foliar application of both phenylalanine and tryptophan in combination has also been shown to lead to an increase in total phenolic compounds present within the plant [41–43, 48]. The ability of aromatic amino acids to improve plant yield and defend against harsh environmental conditions may be related to the antioxidant properties of these phenolic compounds.

#### Tryptophan

In lupines (*Lupines terms L*.), foliar application of tryptophan increased overall growth and levels of several key chemical components*,* including alkaloids and phenolic compounds. Physiological measures of growth increased, such as plant height and the mass and yield of seeds, alongside increases in key chemical contents of the plant, such as total nitrogen, crude protein, total soluble sugars, total alkaloids, and total phenolic compounds [48]. Smaller increases were seen in the levels of ascorbic acid.

#### Tyrosine

Few studies have been published looking at the effect on the yield of tyrosine as a foliar spray. In one study, tyrosine was shown to increase total phenolic content in one species of mint, M. piperita 'Swiss', when applied at a concentration of 100 mg L^-1^, compared to water-sprayed controls. However, this was not found to be a significant increase [39].

Tyrosine has been used as part of protein mixtures applied to leaves, with no indication as to the role that tyrosine may play within the mixture [49]. Tyrosine may, however, have beneficial applications as a soil-based fertiliser [50].

### Acidic amino acids

As has already been shown for some of the aliphatic and aromatic amino acids, foliar application of aspartic acid also improves a plant’s antioxidant defence system. However, unlike most of the previous examples, aspartic acid has not been shown to increase levels of polyphenols in order to elicit this effect. Glutamic acid improves the efficiency of photosynthesis.

#### Aspartic acid

Aspartic acid has been shown to alleviate osmotic stress caused by water salinity in tomato plants (*Solanum lycopersicum*), with this effect being linked to the antioxidant properties of the amino acid as well as the ability of this amino acid to act as an osmoregulatory compound [51]. It has also been used to reduce the effect of oxidative stress caused by cadmium poisoning in water supplies from contaminated sources [52, 53]. The same effect is also seen for foliar application of proline [53].

#### Glutamic acid

Glutamic acid was shown to have a positive impact on growth and fruit quality in grapes, with increased chlorophyll levels, leaf size, and fruit yield. Higher concentrations of nitrogen, potassium, and phosphorus were also found when compared to control plants treated with water [54]. Similar effects of increased chlorophyll levels and rates of photosynthesis have been observed for hawthorn (*Crataegus monogyna*) and Chinese chives (*Allium tuberosum*). It has been posed that glutamate improves electron transfer within photosystem II (PSII), a protein mediated complex responsible for generating energy for the plant, but the mechanism by which this effect occurs is unclear [55, 56].

Time course studies of isotopically labelled glutamate applied to bentgrass leaves (*Agrostis capillaris*) at a concentration of 0.01 mol L^−1^ showed that glutamate is utilised in the leaf as a precursor for *gamma-*aminobutyric acid (GABA) and proline synthesis. These metabolites are known to play a role in plant stress adaptation [57]. This study also concluded that glutamate enters the plant leaf whole, being metabolised within plant cells rather than being metabolised to ammonia on the leaf surface. No control experiments were reported for this study, however.

### Basic amino acids

The effect of foliar application of arginine is very different between plant species. In some plant species, arginine induces an increase in chlorophyll levels, which improves the rate of photosynthesis in a similar way to glutamic acid. Histidine and lysine, by contrast, are both able to form complexes with metals to help transport these metals into plants.

#### Arginine

Studies of arginine applied to leaves showed it to be a poorer growth stimulant than urea but also clarified that the two are metabolised within plants in different ways [58]. When applied to grapevines, no significant difference was seen from the control for measure of pH or total anthocyanins. However, total flavanol levels and proline concentration both decreased [40]. Decreases in flavanols suggest that basic amino acids are metabolised in plants through a different pathway to aromatic amino acids [41–43].

When applied to tomato plants, arginine has been shown to significantly improve plant growth and had a positive effect on total fruit production and chlorophyll content, though no significant effect was seen on the average mass of individual fruits [59].

#### Histidine and lysine

Histidine and lysine have been most extensively studied as their chelates with metals rather than as free amino acids. Complexes of histidine and lysine with metal ions have been shown to improve the resistance of plants to increased salinity and modify their nitrogen content in some tomato cultivars when compared to glycine as a control [60].

Other studies have looked at the corresponding zinc complexes in addition to complexes with lysine [61]. 

The complex with lysine was the most effective foliar spray, with several cultivars of onion (*Allium cepa*) experiencing improved growth, bulb size, and pyruvic acid content, potentially as a consequence of changes in nitrogen metabolism [62]. The application of unchelated lysine did increase both the number and size of leaves but had no effect on nitrogen concentration in the bulb, indicating changes to nitrogen metabolism that resulted from the presence of zinc [63].

### Sulfur-containing amino acids

Both cysteine and methionine are able to reduce the adverse effects of increased salinity in the plant growth medium but exert their influence through different pathways [64].

#### Cysteine

Cysteine has been shown to reverse the negative effects of drought conditions, increasing the fresh weight of roots, root length, and chlorophyll concentration in one maise cultivar, and shoot length, total free amino acids, total phenolics, and free proline contents in a different cultivar [64]. Increased growth, in terms of plant height and yield, has also been noted when cysteine is applied to plants growing under normal conditions [59, 65, 66].

#### Methionine

Methionine, used in conjunction with proline and glutamine and when applied with tryptophan, was shown to reverse the negative effects of salinity in tomato plants. Of particular interest, the study showed that unlike with glycine betaine [Sect.  2.1], the effect is not thought to be mediated by Na^+^ ion concentrations in the leaf but is instead due to an accumulation of soluble sugars [67, 68].

### Amidic amino acids

The effect of foliar application of the amidic amino acids follows the trend of many other amino acids in causing an increase in chlorophyll concentration, which may increase plant growth and yield. Notably, aspartate and glutamate are metabolites of these amino acids and may be responsible for some of the observed effects after foliar application of the corresponding amides.

#### Asparagine

Asparagine, as with many of the other amino acids previously mentioned, produces significant improvements in plant growth after foliar application as a spray to cress (*Lepidium sativum*), including an increase in chlorophyll concentration (both chlorophyll a and b) and leaf nitrogen and phosphorus levels [69]. In increased salinity environments, asparagine was shown to reduce sodium ion concentrations in leaves of maise while increasing levels of phosphorus, calcium, and potassium ions present in the roots [70].

#### Glutamine

Glutamine has been shown to increase height, fresh and dry weight, and chlorophyll concentration of a number of plants, but to a lesser extent than glycine [21, 71]. However, its effect when applied to the roots through the soil is more pronounced [20].

### Hydroxylic amino acids

Serine and threonine have not been extensively examined and do not appear to yield a significant improvement in plant growth.

In one study, serine inhibited plant growth to a similar extent to alanine, whereas threonine showed no significant difference from a control group [16].

Threonine has been used as a ligand for zinc ions in the same manner as histidine and lysine and did increase the number of leaves and leaf area in one cultivar of onion but not the other, and was less effective than Zn-Lys [63].

## Conclusion

It is clear that the foliar application of some, but not all, of the twenty main proteinogenic amino acids is beneficial for the growth of a range of plant species. While some mechanisms are proposed with genetic explanations, many do not have proposed mechanisms (Table 1).

**Table 1:** Table summarising the effect on growth and the proposed mechanisms for the foliar application of the twenty proteinogenic amino acids covered in this review

	Amino acids	Summary of effect on growth	Proposed mechanisms
Aliphatic	Alanine (Ala)	Mostly studied in mixtures • Negative effect on *Eustoma grandilorum* growth • Positive effect on quality of Fuji apples	Contributes to plant’s antioxidant defence system through promotion of polyphenolic compounds—mechanism for this not given
	Glycine (Gly)	Significant variance in effects based on plant species • No effect on maize • At low concentrations, improves growth of lettuce, sweet basil and *Eustoma grandilorum*	Chlorophyll and carotenoid concentrations increase in leaves. Glycine is a precursor for chlorophyll A synthesis, which may be related to its mode of action
	Isoleucine (Ile)	Limited studies on the branched-chain amino acids • Increased root density without change to root length or shoot weight • Similar growth improvements to urea soil application	Improved shoot density is suggested to be related to plant growth regulators, not just the presence of additional nitrogen
	Leucine (Leu)		
	Valine (Val)		
	Proline (Pro)	• Positive effect on plant growth • Mitigates the effects of heat stress, high salinity, and heavy metal-induced stress	An increase in Chlorophyll content was observed. An increase in polyphenolic compounds was observed. Proline may improve nitrogen fixation in the roots, leading to increased amino acid and chlorophyll synthesis.—no specific mechanism for this is given
Aromatic	Phenylalanine (Phe)	• Improves quality of wine grapes through increased levels of phenolic compounds	Increased phenolic compound concentration improves antioxidant activity. The increase in phenolic compounds is due to phenylalanine metabolism leading to increased abscisic acid (ABA) levels, which upregulate genes in the phenolic synthesis pathway
	Tryptophan (Trp)	• Increased overall growth in lupines • Increased plant height, mass, yield of seeds, total nitrogen, crude protein, total soluble sugars, alkaloids and phenolic compounds • Small increase in ABA	The small increase in ABA suggests a different mechanism to Phe, but it is also related to reducing oxidative stress through the production of compounds which can act as antioxidants
	Tyrosine (Tyr)	Limited studies on Tyrosine • Increased total phenolic content in M. piperita ‘Swiss’ mint	Contributes to plant’s antioxidant defence system through promotion of polyphenolic compounds – mechanism for this not given
Acidic	Aspartic acid (Asp)	• Mitigates osmotic stress in tomato plants caused by water salinity • Also reduces oxidative stress from cadmium poisoning • Does not cause an increase in polyphenolic compounds	Acts as an osmoregulatory compound and directly as an antioxidant
	Glutamic acid (Glu)	• Improved chlorophyll levels, leaf size, and fruit yield in grapes • Increased chlorophyll levels and photosynthesis rates in hawthorn and Chinese chives	May improve electron transfer within photosystem II. Shown to act as a precursor for gamma-aminobutyric acid (GABA) in proline synthesis. These are both involved in plant stress adaptation
Basic	Arginine (Arg)	• Increased chlorophyll levels • Improved plant growth and fruit quality in tomatoes • Decrease in total flavanol levels and proline concentration	Different pathway to aromatic amino acids and to urea. No specific mechanism has been proposed
	Histidine (His)	Mainly studied as chelates with metals rather than free amino acids • Improved salinity resistance in some tomato cultivars	No indication of mechanism found
	Lysine (Lys)		
Hydroxylic	Serine (Ser)	Very few studies on these compounds One study showed: • Serine inhibited plant growth similarly to alanine • threonine showed no significant difference from the control group	No indication of mechanism found
	Threonine (Thr)		
Sulfur-containing	Cysteine (Cys)	• Improved salinity and drought resistance • Increased root and shoot growth, chlorophyll concentration, and phenolic compounds in maize	No indication of mechanism, though may be related to the mechanism of others that increase chlorophyll concentration
	Methionine (Met)	• Improved salinity resistance when used in conjunction with other amino acids	Mechanism believed to be related to the accumulation of soluble sugars
Amidic	Asparagine (Asn)	• Increased chlorophyll concentration, leaf nitrogen and phosphorus levels • Can reduce the effects of high-salinity environments	In a high-salinity environment it reduces sodium ion concentrations in maize leaves while increasing phosphorus, calcium, and potassium ion levels in the roots. No mechanism for chlorophyll increase given
	Glutamine (Gln)	• Increased height, fresh and dry weight, chlorophyll concentration (not as well as glycine) • More pronounced effect when applied as soil treatment	No indication of mechanism found

**Fig. 3:**
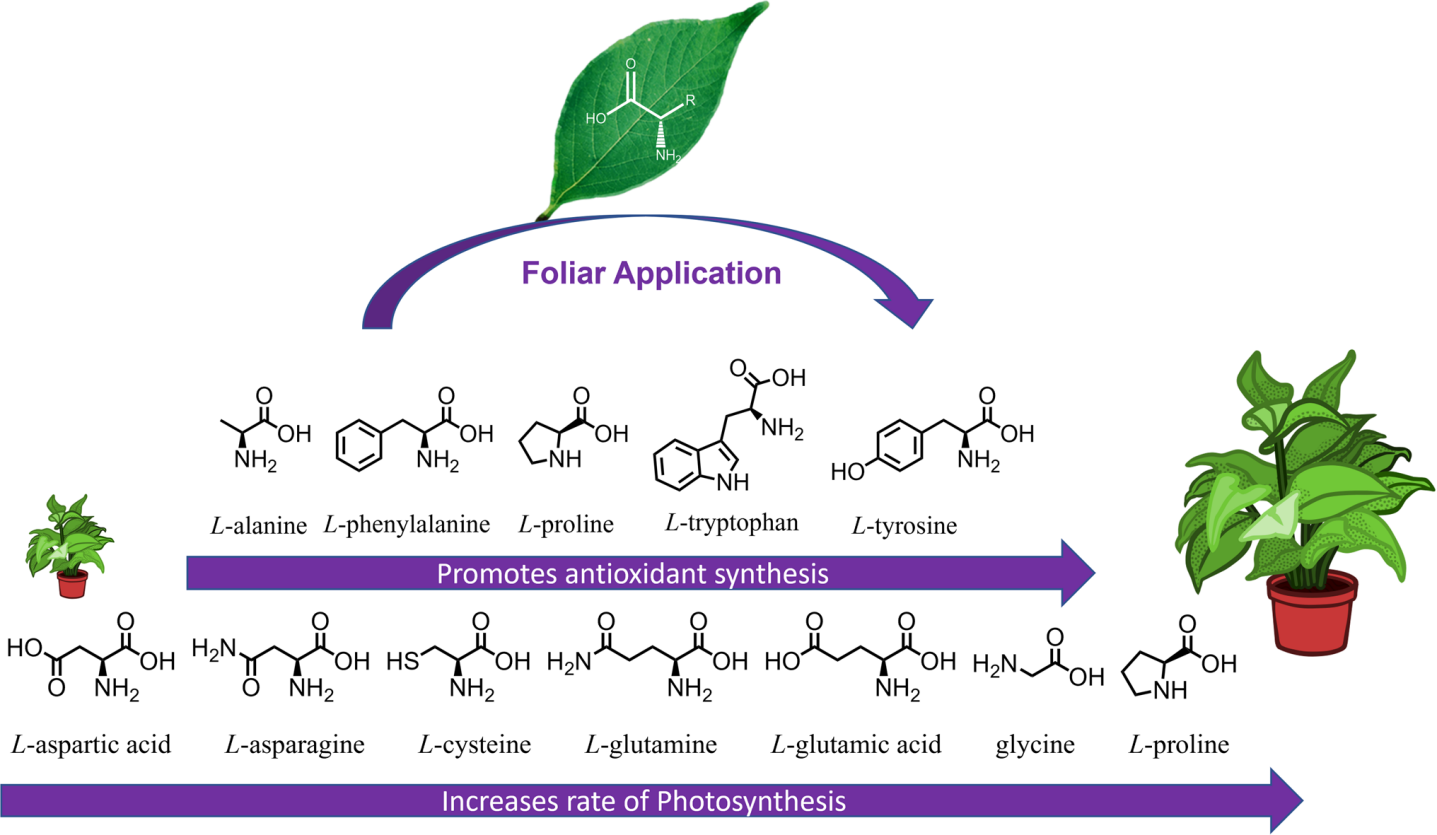
Two main modes of action are seen in the literature for the foliar application of amino acids. Some increase levels of antioxidants, and some increase the rate of photosynthesis by promoting chlorophyll synthesis or (in the case of glutamic acid) by improving electron transfer in PSII

These benefits are typically attributed to increasing resistance to stress conditions or to promoting the synthesis of chlorophyll (Fig. 3) [36, 37, 52, 53]. However, in some cases, the control experiments were either not documented or insufficient to isolate the amino acid as the beneficial agent. When significant errors of measurement for some metrics, such as dry mass, are factored in, it becomes clear that more research is needed to verify these hypotheses. In addition, other routes by which beneficial effects may be mediated have been proposed, including amino acids providing a direct supply of “building blocks” for protein synthesis, thereby reducing the metabolic energy required for the nascent amino acid synthesis [16].

Both chlorophyll synthesis and resistance to stress involve the production of beneficial compounds to the plant. In the latter case, the generation of polyphenolic compounds after foliar application of phenylalanine is accompanied by the upregulation of specific genes. It may be of interest to examine whether the activity of these genes can be linked to the applications of other amino acids.

**Fig. 4:**
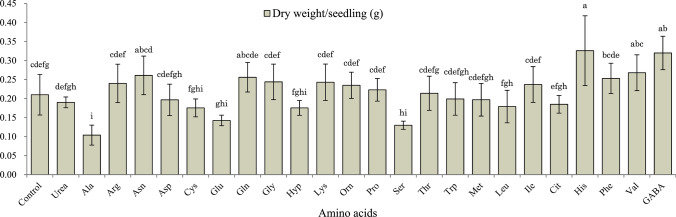
Comparative study of foliar application of 23 amino acids and urea to Eustoma grandiflorum. Results show some difference in the dry weight of seedlings between treatments, but large error bars make the significance of these effects questionable. Reprinted from [16], Copyright (2015), with permission from Elsevier

Adapting the use of amino acids for the agricultural market requires more research into the workings of individual amino acids across a wider range of plant species. For several of the major amino acids there is a scarcity of available data. In some cases, this may be due to poor results from initial studies, but without comparative data, it cannot be confirmed what effect specific amino acids have when applied as a foliar spray. Where comparative studies have been done, such as with *Eustoma grandiflorum*, error bars are often too large for any significant effect to be ascertained (Fig. 4) [16].

The effect of the foliar application that proves most significant is dependent on both the specific amino acid used and the species or genotype of the plant to which it is applied to [50]. However, the amount of amino acid given to each plant is often difficult to assess from the concentration of the treatment solution, as given in many studies, since the output speed and surface area of the plant to be sprayed significantly affect the dosing. A useful additional measurement for those wishing to apply this data in an agricultural setting would be litres per hectare, which indicates the volume of output, plant coverage and spraying speed.

To design future synthetic biostimulants based on naturally occurring amino acids or to produce the most cost-effective amino acid mixtures, it is necessary to understand in more detail how different individual compounds react within a plant and if these modes of action can be complementary to or hinder each other’s effects. For example, aromatic amino acids increase the levels of polyphenols, whereas proline and aspartic acid quench reactive oxygen species. Both increase resistance to oxidative stress and promote growth and yield through different modes of action [36, 72].

Overall, there is a necessity for more rigorous and comparative research to fully elucidate the specific roles and optimal applications of individual amino acids in plant growth, which will be crucial for developing effective biostimulants and advancing agricultural practices.

## Supplementary Information

Below is the link to the electronic supplementary material.

Supplementary Material 1.

## Data Availability

No datasets were generated or analysed during the current study.
